# Non-insulinoma pancreatogenous hypoglycemia syndrome due to nesidioblastosis following bariatric Roux-en-Y gastric bypass

**DOI:** 10.1093/jscr/rjad050

**Published:** 2023-02-17

**Authors:** Josephine A Gottsponer, David L Baker, Tamara Osborn, Martha M Estrada, Emmanouil Giorgakis, Lyle J Burdine, Raj B Patel

**Affiliations:** College of Medicine, University of Arkansas for Medical Sciences, Little Rock, AR, USA; Department of Surgery, University of Arkansas for Medical Sciences, Little Rock, AR, USA; Department of Surgery, University of Arkansas for Medical Sciences, Little Rock, AR, USA; Department of Surgery, University of Arkansas for Medical Sciences, Little Rock, AR, USA; Department of Surgery, University of Arkansas for Medical Sciences, Little Rock, AR, USA; Department of Surgery, University of Arkansas for Medical Sciences, Little Rock, AR, USA; Department of Surgery, University of Arkansas for Medical Sciences, Little Rock, AR, USA

## Abstract

A 56-year-old woman with past medical history significant for bariatric Roux-en-Y gastric bypass 3 years prior presented for evaluation of an 8-month history of severe hypoglycemia relieved by intake of carbohydrates associated with syncopal episodes. Inpatient workup revealed endogenous hyperinsulinemia concerning for insulinoma vs. nesidioblastosis. She successfully underwent pancreaticoduodenectomy (Whipple procedure), and pathology report confirmed scattered low-grade intraepithelial neoplasia within the pancreatic parenchyma consistent with nesidioblastosis. The patient has had satisfactory control of glucose levels 30 days out from surgery.

## INTRODUCTION

We present a case of adult-onset noninsulinoma pancreatogenous hypoglycemia syndrome determined to be caused by nesidioblastosis 3 years status-post Roux-en-Y gastric bypass. This 56-year-old presented with several months of symptomatic hypoglycemia and syncope and underwent extensive diagnostic testing before being treated surgically with satisfactory results.

## CASE REPORT

A 56-year-old female was admitted for evaluation of suspected insulinoma after outside imaging showed a pancreatic mass. She had been experiencing 8 months of symptomatic hypoglycemia associated with syncope and relieved by intake of carbohydrates. Medical history included DM-II without medication requirement since 2018 and bariatric surgery 3 years prior.

Physical exam revealed a woman with a body mass index of 29 with no obvious signs of pathologic processes; abdominal examination was negative for distention, palpable masses and tenderness. Labs revealed mild anemia (Hct 33.9%), hypokalemia (3.4 mmol/L) and hyperglycemia (114 mg/dl). Her c-peptide and insulin levels were both elevated at 4.71 ng/ml and 27.9 μU/ml. Her HbA1c was 4.9%.

Esophagogastroduodenoscopy and endoscopic ultrasound were performed and showed that the gastrojejunostomy anastomosis from her prior bariatric surgery was quite large and could have been contributing to her glycemic lability. These studies were otherwise unremarkable and revealed no pancreatic masses concerning for insulinoma. Selective arterial calcium stimulation test (SACST) resulted in increased insulin secretion to four times normal levels localized to the gastroduodenal artery. In a 72-h fasting glucose test, her glucose level stayed consistently above 60. Following this testing, it was determined that the patient required no urgent intervention and was discharged for outpatient management with octreotide and low-carbohydrate diet. The possibility of future surgical intervention was also discussed with the patient should medical management prove inadequate.

Two weeks later, the patient presented to the emergency department again for evaluation of a severe hypoglycemic episode with a blood glucose level of 44. Computed tomography-abdomen on this admission again showed no enhancing pancreatic mass but did show intrahepatic biliary ductal dilation to 1 cm as well as pancreatic ductal dilation to 4.3 mm concerning for a pancreatic head mass. The primary differential at this time was insulinoma vs. nesidioblastosis, and on Day 4 of this admission, the patient was transferred to the surgical oncology team for management via a Whipple procedure.

The abdomen was opened through midline and right subcostal incisions. There was no discrete pancreatic mass appreciated on gross examination. Major interventions included cholecystectomy, partial pancreatectomy, including the head of the pancreas, with associated duodenectomy and resection of the stomach remnant. The previously existing Roux-en-Y was also partially reconstructed with the addition of the hepaticoduodenostomy and pancreaticojejunostomy.

The pathology report on excised tissues revealed mild chronic cholecystitis with one benign lymph node associated with the cholecystectomy specimen as well as scattered low-grade intraepithelial neoplasia within the pancreatic parenchyma. All margins were negative for malignancy, and all 43 lymph nodes were benign. This report, as well as the combination of her negative 72-h fasting glucose test and positive SACST, more strongly supported the diagnosis of nesidioblastosis. Thirty days postoperatively, the patient’s glucose levels had remained within the normal range.

## DISCUSSION

Nesidioblastosis is a cause of hypoglycemia much more common in neonates but that has occasionally been identified as a new diagnosis in adults. In contrast to insulinoma, which is a proliferation of β-islet cells in a discrete, localized pancreatic mass, nesidioblastosis is characterized by diffuse proliferation of enlarged β-islet cells interspersed throughout the pancreas [1, 2].

Diagnosis of nesidioblastosis often begins with ruling out insulinoma. Laboratory studies, including high insulin and c-peptide levels and low β-hydroxybutyrate, indicate endogenous hyperinsulinemia. Imaging studies often show no discrete masses; if masses are seen, they may be multiple due to the diffuse nature of the pathology. SACST is also a useful diagnostic tool. This test works by injection of calcium gluconate followed by measurement of insulin secretion in several arteries surrounding the pancreas; a marked increase from baseline in these arteries identifies an area with increased β-islet cell activity [3]. Positive results in multiple locations both confirm pancreatic pathology as the origin of hypoglycemia while also leading away from the differential of insulinoma due to the lack of a single area of localization [4, 5]. Interestingly, in this patient, SACST was positive in only the head of the pancreas, represented as increased insulin levels in the gastroduodenal artery. Therefore, an SACST that is positive but not diffusely so should not rule out a diagnosis of nesidioblastosis; several foci of proliferation may occur in close enough proximity to one another to yield what appears to be a localized pathology. This result is superficially more suggestive of insulinoma but is not definitive. Thorough history-taking is also crucial; many patients with nesidioblastosis present with postprandial hypoglycemia, whereas insulinoma will cause a constant state of hypoglycemia, particularly in the early morning, due to continuous release of excess insulin regardless of fed or fasting state [2].

There have been several reports linking adult-onset nesidioblastosis with history of bariatric surgery, specifically Roux-en-Y gastric bypass, which this patient had undergone 3 years prior. The proposed mechanism revolves around the idea that the physiologic effects of gastric bypass lead to an increase in β-islet cell trophic factors. One study hypothesizes that the increase in glucagon-like peptide-1 often seen in dumping syndrome could promote increased β-islet cell mass, resulting in nesidioblastosis [6].

Treatment ranges from dietary management to surgical intervention. Because many patients present with primarily postprandial hypoglycemia, dietary management with a low-carbohydrate diet, similar to the management of reactive postprandial hypoglycemia, is adequate for many [7]. For refractory cases, surgical intervention with partial pancreatectomy is the mainstay of treatment and results in satisfactory reduction in symptoms for most patients. There have also been reports describing medical management with somatostatin analogs such as pasireotide [8]. In this case, failure of this medication to adequately control the patient’s hypoglycemia, as well as cost considerations, led to the conclusion that surgical intervention was her preference for treatment. It is also notable that, because this patient’s SACST was localized to the head of the pancreas, a Whipple procedure was adequate for resolution of symptoms with lower post-operative morbidity compared with total pancreatectomy that may be required in patients with more diffuse proliferation of β-islet cells.

## CONFLICT OF INTEREST STATEMENT

None declared.

## FUNDING

None.

## DATA AVAILABILITY

Further data may contain identifiable information and is not available to the public.
